# Incorporation of Copper-Doped Mesoporous Bioactive Glass Nanospheres in Experimental Dental Composites: Chemical and Mechanical Characterization

**DOI:** 10.3390/ma14102611

**Published:** 2021-05-17

**Authors:** Danijela Marovic, Håvard J. Haugen, Visnja Negovetic Mandic, Matej Par, Kai Zheng, Zrinka Tarle, Aldo R. Boccaccini

**Affiliations:** 1 Department of Endodontics and Restorative Dentistry, School of Dental Medicine, University of Zagreb, 10000 Zagreb, Croatia; vnegovetic@sfzg.hr (V.N.M.); mpar@sfzg.hr (M.P.); tarle@sfzg.hr (Z.T.); 2 Department of Biomaterials, Institute of Clinical Dentistry, University of Oslo, 0455 Oslo, Norway; h.j.haugen@odont.uio.no; 3 Department of Materials Science and Engineering, Institute of Biomaterials, University of Erlangen-Nuremberg, 91058 Erlangen, Germany; kai.zheng@fau.de (K.Z.); aldo.boccaccini@fau.de (A.R.B.)

**Keywords:** dental, resin composites, copper, bioactive glass, mesoporous, nanoparticles

## Abstract

Experimental dental resin composites incorporating copper-doped mesoporous bioactive glass nanospheres (Cu-MBGN) were designed to impart antibacterial and remineralizing properties. The study evaluated the influence of Cu-MBGN on the mechanical properties and photopolymerization of resin composites. Cu-MBGN were synthesized using a microemulsion-assisted sol–gel method. Increasing amounts of Cu-MBGN (0, 1, 5, and 10 wt %) were added to the organic polymer matrix with inert glass micro- and nanofillers while maintaining a constant resin/filler ratio. Six tests were performed: X-ray diffraction, scanning electron microscopy, flexural strength (FS), flexural modulus (FM), Vickers microhardness (MH), and degree of conversion (DC). FS and MH of Cu-MBGN composites with silica fillers showed no deterioration with aging, with statistically similar results at 1 and 28 days. FM was not influenced by the addition of Cu-MBGN but was reduced for all tested materials after 28 days. The specimens with 1 and 5% Cu-MBGN had the highest FS, FM, MH, and DC values at 28 days, while controls with 45S5 bioactive glass had the lowest FM, FS, and MH. DC was high for all materials (83.7–93.0%). Cu-MBGN composites with silica have a potential for clinical implementation due to high DC and good mechanical properties with adequate resistance to aging.

## 1. Introduction

Conventional restorative materials have several drawbacks, with one of the greatest being the occurrence of caries surrounding them, the so-called secondary caries [1]. Secondary caries is the major cause for restoration replacement and causes a significant workload escalation and economic burden [2].

The issue of secondary caries has enticed our research group to develop remineralizing bioactive materials with the addition of amorphous calcium phosphate (ACP) [3,4,5,6] and 45S5 bioactive glass (BG) fillers [7,8,9,10,11]. The supersaturated calcium and phosphate ion concentrations released by ACP resin composites have been shown to induce hydroxyapatite formation and remineralization [12]. Silica nanofillers (SiO_2_) have been shown to enhance apatite formation on the surface of both ACP and BG composites [4]. However, the amorphous structure and solubility of ACP fail to provide strong mechanical properties of resin composites [3,4,5]. Similar behavior was demonstrated by BG resin composites, whose mechanical properties and degree of conversion (DC) deteriorated in a dose-dependent manner with the increase in BG ratio [8,10]. None of the investigated bioactive fillers, i.e., neither ACP nor BG, were silanized.

In commercial composites, the silane coupling layer is a bifunctional molecule that simultaneously creates a covalent Si–O–Si bond to the filler particles and copolymerizes with the methacrylate groups in the resin phase. The synergy between the filler and the resin increases the mechanical strength of the composite material by arresting crack propagation [13]. On the other hand, silanization hinders the ion release from bioactive fillers and is commonly considered undesirable for maintaining the required filler reactivity [14,15,16].

To counteract the abovementioned issue and avoid silanization of bioactive fillers, mesoporous bioactive glass nanospheres seem to be a suitable alternative. Mesoporous particles have a higher surface area than dense spherical ones due to a porous structure. Theoretically, low-viscosity resin monomers could penetrate pores and remain mechanically interlocked in their position after polymerization [17]. Therefore, the necessity of chemical bonding through silanization could be circumvented because of the micromechanical interlocking of resin and bioactive mesoporous particles [18]. Besides, greater reactive surface of mesoporous bioactive particles provides enhanced bioactivity compared to conventional bioactive glass [19]. This fact reduces the need for high amounts of bioactive glass in resin composites to achieve the remineralizing effect.

Mesoporous bioactive glass is currently in the spotlight of regenerative medicine. In addition to dense bioactive glass particles possessing the ability to release calcium, phosphorus, and silicon ions [20], mesoporous particles can act as carriers of therapeutic ions, such as copper, silver, or zinc [21,22,23,24,25].

Zheng et al. developed copper-doped mesoporous bioactive nanospheres (Cu-MBGN) by a sol–gel method using a Cu/L–ascorbic acid complex as the precursor of Cu [24]. This method enabled better particle dispersion, which represents a common problem of nanosized particles [26]. Synthesized spherical particles were 100–300 nm with 2–10 nm pore size [24]. Cu-MBGN are primarily synthesized for biomedical applications, particularly osteogenic regeneration [24]. Application of Cu-MBGN caused a reduction in bacterial viability of *Staphylococcus aureus*, *Escherichia coli*, and *Staphylococcus epidermidis* [19,27,28] as well as hydroxyapatite formation upon exposure to simulated body fluid [19,24].

If added to conventional resin monomers and silanized inert micro- and nanofillers, the end product could be a multifunctional dental composite with highly desirable properties, namely antibacterial, ion-releasing/remineralizing, and improved mechanical strength. To our knowledge, no similar composite materials have been synthesized and investigated so far.

This study aimed to examine the influence of the addition of Cu-MBGN with proven antibacterial effect on selected mechanical and curing properties of experimental resin composites. Two strategies were tested in this study:

1. Bimodal approach: A material containing only Cu-MBGN fillers and inert silanized microfillers was compared to control materials. For the inert control material, a composite containing inert silica nanofillers and inert microfillers was used. The bioactive control consisted of conventional bioactive glass 45S5 and inert microfillers.
2. Trimodal approach: Similar to commercial materials, three types of fillers were used, namely inert silanized microfillers, inert silanized silica nanofillers, and various amounts of unsilanized Cu-MBGN fillers. Inert and bioactive control materials had identical composition as in the bimodal approach but with the total filler amount adjusted to 70 wt %.

The first null hypothesis of the bimodal strategy was that the addition of Cu-MBGN would not influence the flexural strength (FS), flexural modulus (FM), Vickers microhardness (MH), and DC in comparison to inert silica fillers or commercial bioactive fillers. The second null hypothesis of the trimodal strategy was that the combination of silica and increasing amounts of Cu-MBGN would not improve the tested properties (FS, FM, MH, and DC) over Cu-MBGN alone.

## 2. Materials and Methods

### 2.1. Synthesis of Cu-MBGN

Cu-MBGN were synthesized using a microemulsion-assisted sol–gel method as reported in our previous work [24]; the detailed synthesis procedures can be found in the literature. The chemical composition of Cu-MBGN was 85.4SiO_2_–10.2CaO–4.4CuO (in mol %) as reported in the literature [24].

### 2.2. X-ray Diffraction (XRD)

XRD patterns of Cu-MBGN and 45S5 BG (Schott AG, Mainz, Germany) powders were recorded at room temperature with a Panalytical Aeris powder diffractometer (Malvern Panalytical, Malvern, UK) using CuKα_1,2_ radiation.

### 2.3. Scanning Electron Microscope (SEM)

The commercial 45S5 BG and Cu-MBGN powders were sputter-coated with 5 nm Au/Pd layer (90% Au/10% Pd), evaporated with argon plasma at 6 kV using the Precision Etching Coating System (Model 682, Gatan Inc., Pleasanton, CA, USA).

FE-SEM images of prepared samples were taken with a scanning electron microscope JSM7000F (JEOL Ltd., Tokyo, Japan) linked to the energy-dispersive X-ray analyzer EDS/INCA 350 (Oxford Instrument, Abingdon, UK).

### 2.4. Mixing of Experimental Resin Composites

Bisphenol A–glycidyl methacrylate (BisGMA) and triethylene glycol dimethacrylate (TEGDMA) were purchased from Merck, Darmstadt, Germany. An identical resin matrix containing 60:40 weight ratio of BisGMA: TEGDMA with a photoinitiator system (camphorquinone (0.2 wt %; Merck) and ethyl-4-dimethylamino benzoate (0.8 wt %; Merck)) was used for all materials. Resin was heated to 60 °C prior to admixture of fillers.

Four types of fillers were used in this study, as shown in Table 1.

The materials were mixed in the absence of blue light using an asymmetrical centrifugal mixer (Speed Mixer TM DAC 150 FVZ, Hauschild & Co KG, Hamm, Germany) at gradually increasing speed up to 2700 rpm.

Eight experimental resin composites were prepared, divided into two groups:

1. Group testing the bimodal approach with 65 wt % total filler load and
2. Group testing the trimodal approach with 70 wt % total filler load used for investigating 1, 5, and 10 wt % Cu-MBGN composites with silica fillers.

Each group contained both inert control (silica and microfillers; 10-Si and 15-Si) and bioactive control (45S5 BG and microfillers, 10-BG and 15-BG) in adequate amounts corresponding to the total filler load (Table 2).

### 2.5. Flexural Strength and Modulus

FS and FM were measured according to ISO/DIN 4049:1998 using the three-point bending test [29]. Custom-made stainless steel mold with an opening and dimension of 16 × 2 × 2 mm^3^ was filled with the composite paste, covered with polyester strips on both sides to prevent oxygen-inhibited polymerization layer formation, and pressed with a weight to extrude excess material. The specimens were light-cured three times on each side with overlapping exposures, i.e., six times in total. The light guide of the curing unit was always in direct contact with the polyester strip. Composite specimens were photopolymerized for a total of 120 s (6 × 20 s) with a light-curing unit (Bluephase PowerCure, Ivoclar Vivadent, Schaan, Liechtenstein, 950 mW/cm^2^). The upper side that was polymerized first was marked, and the specimens were stored in saline solution in the dark in an incubator at 37 ± 1 °C for 1 or 28 days. Ten specimens were prepared per material and time point.

After the designated time, the specimens were removed from the saline solution, dried, and tested immediately with the side that was irradiated first facing the jig. The customized universal testing machine Ultratester (Ultradent, Salt Lake City, UT, USA) was used at a 1 mm/min crosshead speed until specimen failure.

### 2.6. Vickers Microhardness

Vickers microhardness (MH) was measured using Vickers hardness testing machine CSV-10 (ESI Prüftechnik GmbH, Wendlingen, Germany). The specimens prepared for the three-point bending test and aged for 1 or 28 days in saline solution at 37 °C were polished to remove the surface resin-rich layer with 4000 grit silicon carbide paper and 0.05 µm aluminum oxide slurry. The measurements were made with a 100 g load and 15 s dwell time at the specimen surface. Six specimens per material were subjected to five measurements per depth. The data from different measuring points on the same specimen were pooled and treated as a statistical unit.

### 2.7. Degree of Conversion

The measurements were made using FT-Raman spectroscopy (Spectrum GX spectrometer, PerkinElmer, Waltham, MA, USA). The excitation source was a NdYaG laser (1064 nm), kept at 400 mW laser power and resolution of 8 cm^−1^. For each spectrum, 300 scans were recorded.

DC was measured on the top and bottom surfaces of the specimens that were previously stored in saline solution for 1 day (*n* = 5) to determine the mechanical properties. Spectra of the uncured composites (*n* = 5) were acquired correspondingly. Kinetics add-on for MATLAB (Mathworks, Natick, MA, USA) was used for spectral analysis.

DC calculation was performed by comparing the peak heights of aliphatic C–C stretching mode at 1638 cm^−1^ and aromatic C···C band at 1608 cm^−1^ attained from cured and uncured specimens using the following equation:

(1) $$\mathrm{DC}\;\left(\%\right)=\left(1\;-\;\frac{{(1638\;\mathrm{cm}}^{-1}{/1608\;\mathrm{cm}}^{-1})\;\mathrm{after}\;\mathrm{curing}}{{(1638\;\mathrm{cm}}^{-1}{/1608\;\mathrm{cm}}^{-1})\;\mathrm{before}\;\mathrm{curing}}\right)\;\times \;100\;\%\;$$

### 2.8. Statistical Analysis

Mean values of FS, FM, MH, and DC were compared among the experimental materials using one-way ANOVA with Tukey’s post-hoc adjustment for multiple comparisons. Pairwise comparisons (between 1 and 28 days for FS and FM; between 0 and 2 mm for the DC) were compared using a two-tailed *t*-test for independent samples. The *t*-test for independent samples was used for pairwise comparisons between 1 and 28 days for MH; *p*-values lower than 0.05 were considered statistically significant. The statistical analysis was performed using SPSS (version 20, IBM, Armonk, NY, USA).

## 3. Results

### 3.1. X-ray Diffraction

**Figure 1:**
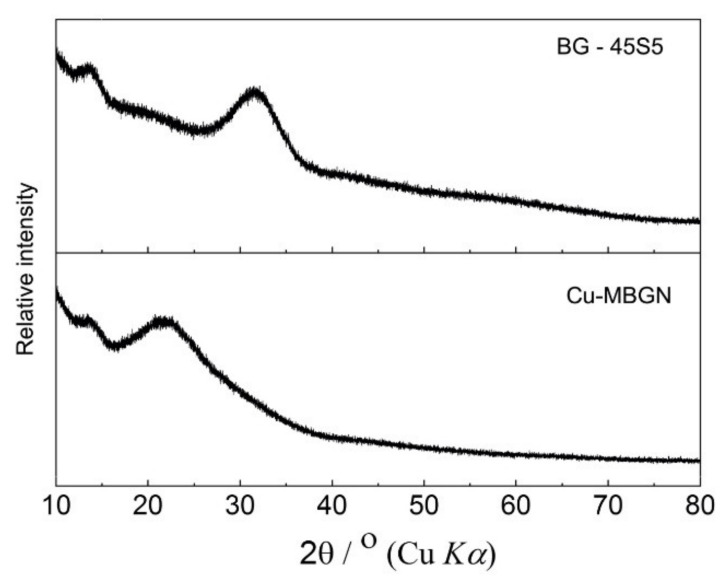
XRD spectra of conventional 45S5 bioactive glass (BG-45S5) and copper-doped mesoporous bioactive glass nanospheres (Cu-MBGN).

Figure 1 shows the amorphous structure of both bioactive glasses used in the study. Cu-MBGN showed a typical XRD pattern of amorphous silicate materials, in which only a broad band located at 2θ = 23° was observed [24].

### 3.2. Scanning Electron Microscopy

**Figure 2:**
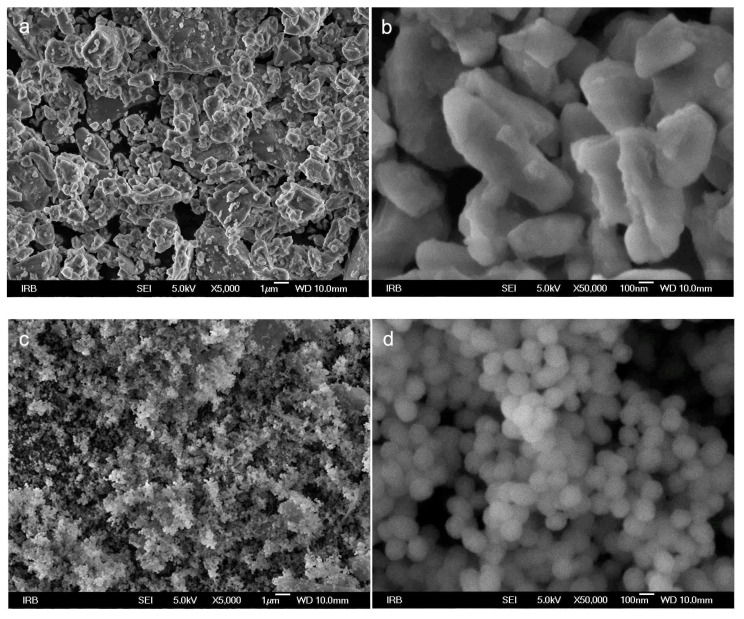
SEM of individual filler particles at 5000× and 50,000× magnification: (**a**,**b**) 45S5 bioactive glass; (**c**,**d**) Cu-MBGN.

Figure 2 shows a typical morphology of bioactive glass used in experimental materials. Conventional 45S5 BG had an irregular structure with a wide dispersion of particle sizes ranging from 200 nm up to 10 µm, with an average of 2–3 µm. Cu-MBGN presented uniform spherical particles that were approximately 100 nm in diameter.

### 3.3. Flexural Strength

**Figure 3:**
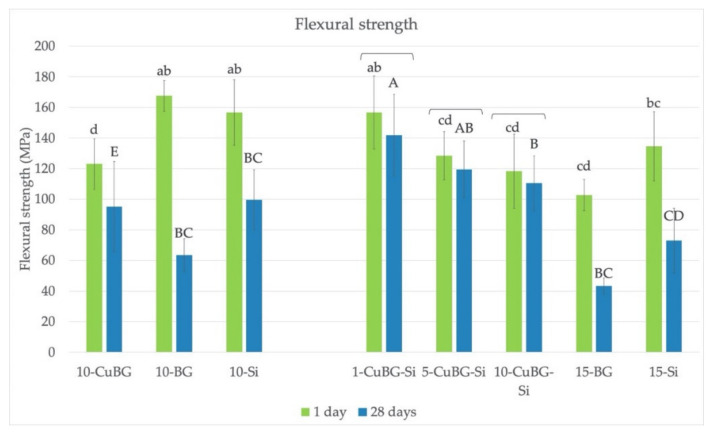
Flexural strength of the tested materials. Lower case letters indicate no statistically significant differences between groups after 1 day. Upper case letters indicate no statistically significant differences between groups after 28 days. Brackets indicate no statistically significant difference within the same material between 1 and 28 days.

The results shown in Figure 3 indicate that 1-CuBG-Si had the highest FS among Cu-MBGN-containing materials, both after 1 day and after 28 days. Control materials 10-BG and 10-Si had similarly high values at 1 day but saw a significant drop after 28 days of exposure to saline solution. The combination of Cu-MBGN and silica also seemed to be beneficial as there was no difference between 1 and 28 days for 1-CuBG-Si, 5-CuBG-Si, and 10-CuBG-Si. On the other hand, materials with Cu-MBGN only (10-CuBG), BG only (10-BG and 15-BG), or silica only (10-Si and 15-Si) did not demonstrate the same stability.

### 3.4. Flexural Modulus

**Figure 4:**
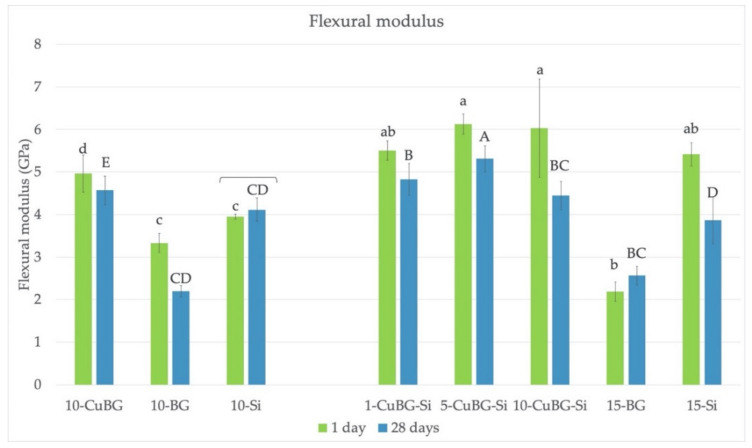
Flexural modulus of the tested materials. Lower case letters indicate no statistically significant differences between groups after 1 day. Upper case letters indicate no statistically significant differences between groups after 28 days. Brackets indicate no statistically significant difference within the same material between 1 and 28 days.

The results are depicted in Figure 4. Again, materials containing both Cu-MBGN and silica had the highest modulus after 1 day, regardless of their amount. However, there was no difference from the inert control 15-Si. After 28 days, 5-CuBG-Si was the material with the highest values. In contrast, materials with 10 and 15% of regular bioactive glass without silica (10-BG and 15-BG) had the lowest modulus.

### 3.5. Microhardness

**Figure 5:**
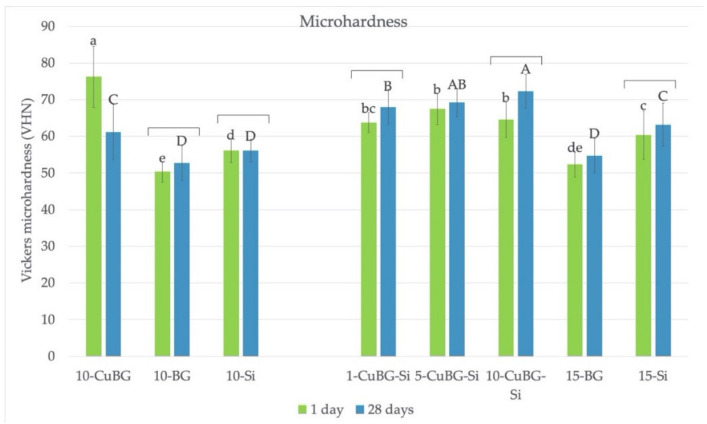
Vickers microhardness (VHN) of the tested materials after 1 and 28 days of exposure to saline solution measured at the top of the specimen. Lower case letters indicate no statistically significant differences between groups after 1 day. Upper case letters indicate no statistically significant differences between groups after 28 days. Brackets indicate no statistically significant difference within the same material between 1 and 28 days.

The MH (Figure 5) was generally equal or higher for CuBG composites than the corresponding controls after the 28 days of exposure to saline solution. Materials 1-CuBG-Si and 10-CuBG-Si had significantly higher MH after 28 days. Material 10-CuBG was the only one with a significant decrease in MH after prolonged contact with the aqueous environment.

### 3.6. Degree of Conversion

**Figure 6:**
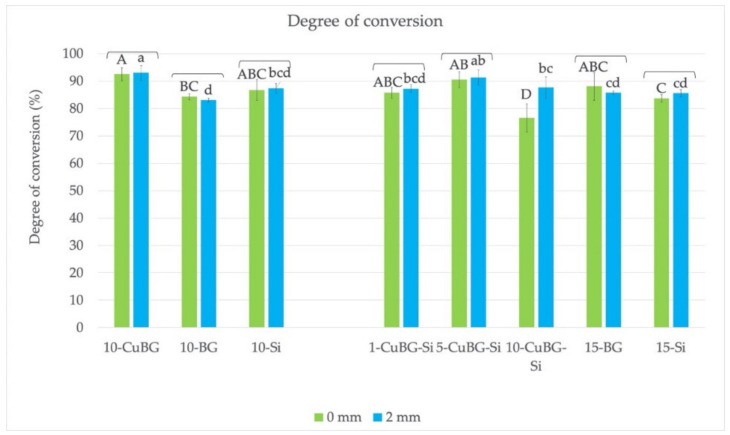
Degree of conversion of the tested materials 1 day after polymerization and exposure to saline solution. Upper case letters indicate no statistically significant differences between groups at the top (0 mm) surface. Lower case letters indicate no statistically significant differences between groups at the bottom (2 mm) surface. Brackets indicate no statistically significant difference within the same material between the top and bottom surfaces.

The results are shown in Figure 6. All the materials, except 10-CuBG-Si, had a very high DC of above 80% at the top (0 mm) and bottom (2 mm) surfaces. There were no differences between the top and bottom surfaces for all materials. The exception was 10-CuBG-Si, which showed the lowest top DC, while the DC at 2 mm was statistically similar to other Cu-MBGN materials with silica.

## 4. Discussion

Incorporation of antimicrobial and remineralizing components into a dental resin composite could be an effective strategy for preventing secondary caries. In this study, experimental dental composites with Cu-MBGN were developed to mitigate this issue. Trimodal approach with the addition of Cu-MBGN combined with silica nanofillers and inert glass microfillers seemed to benefit the durability of their FS and MH, especially after 28 days of exposure to saline solution. Conversely, FS, FM, and MH of the corresponding control materials containing 45S5 bioactive glass with irregular microsized particles were the lowest at the same time point. Both null hypotheses were rejected.

### 4.1. Bimodal Approach

The design of a bioactive dental restorative material is challenging. Demanding oral conditions of repeated masticatory load and constant changes in pH, temperature, chemical erosion, and enzymatic degradation require a mechanically resistant and stable material. On the other hand, bioactive materials interact with living tissue and release certain substances. Thus, material deterioration over time, at least to some extent, is expected. The balance between these opposites is delicate, and the materials should always be tested after intermediate or long-term exposure to an aqueous environment similar to saliva. To minimize unwanted material degradation, stable inert fillers with reinforcing properties were combined with bioactive fillers. Owing to the large reactive surface of Cu-MBGN, bioactivity is expected even when introduced in smaller quantities, allowing five times higher share of inert fillers. In this study, we opted to compare the sole effect of Cu-MBGN particles on the mechanical and curing properties of composite bimodal mixtures to inert and commercial bioactive control before examining the combined effect of Cu-MBGN and silica nanofillers in a trimodal approach.

Bimodal 10-CuBG without silica fillers showed lower FS than the corresponding inert (10-Si) and bioactive control (10-BG). The strength of the resin composites is mainly controlled by the amount of fillers, their size, and the quality of their bond to resin [30]. The mesoporous nanoparticles used here were characterized by a large surface area and small volume but no silanization. The lack of filler/matrix bond most likely interrupted effective stress transfer (between fillers and resin) upon specimen loading. While the addition of silanized fillers improves the FS, the addition of unsilanized fillers reduces it [30]. Similar results were obtained in a study that examined the effect of mesoporous silica nanoparticles on acrylic bone cement [31] and poly(methyl methacrylate) for denture base [32], where the increase in the unsilanized filler load decreased the FS. Conversely, silanized zinc-doped mesoporous silica nanoparticles increased the FS of the experimental dental resins in a simple unimodal filler mixture with a maximum 15 wt % total filler loading [33].

Our SEM analysis showed a significant difference in the shape and size of two bioactive glasses used in the present study. Irregular shape of 45S5 BG is typical for a melt-quench method, while microemulsion-assisted sol–gel method developed by Zheng et al. [24] typically produces spherical particles with diameter of 100–300 nm, as demonstrated in this study. Expectedly, both bioactive fillers showed amorphous structure in XRD analysis. Despite being unsilanized, commercial bioactive glass fillers in 10-BG were significantly larger (4 μm) and thus had a lower contact surface area to resin. Therefore, there were fewer crack initiation sites, which led to a short-term favorable FS in 10-BG. However, the well-known water degradation of 45S5 BG caused the FS of 10-CuBG to surpass the FS of 10-BG after 28 days of exposure to saline solution.

Unlike FS, the FM and MH were enhanced by introducing Cu-MBGN, manifested by the higher values for 10-CuBG than 10-Si and 10-BG. The increase in FM is directly correlated to the filler loading but independent of the interfacial adhesion [30]. Generally, the addition of fillers elevates the FM of the resin matrix because most fillers have greater stiffness than organic polymers [30]. Similarly, an increase in the filler volume and DC improves the MH [34]. The higher filler volume of 10-CuBG compared to that of 10-Si explains our results, similar to other studies [18,31,32].

However, higher filler volume cannot explain the low FM of 10-BG specimens. Commercial BG fillers previously showed a dose-dependent decrease in FM and FS in composites with identical resin matrix and fillers and similar filler amounts as in the present study [8]. High hydrophilicity of 45S5 particles and concomitant water sorption, along with the lack of interfacial bonding and low DC, were identified as culprits for poor mechanical properties. The same dose dependency for FS and FM was seen in this study.

In conclusion, the incorporation of Cu-MBGN in bimodal composite mixtures led to improvement of polymerization, stiffness, and hardness but degradation of strength.

### 4.2. Trimodal Approach

To control the strength reduction caused by the Cu-MBGN addition, further refinement and adaptation of the composite structure was necessary. The majority of contemporary commercial nanofilled composite materials consist of microfillers with a minor share of nanofillers. Smaller particles occupy the space between larger particles and increase the material’s packing density [26]. This approach was also used here to achieve a higher filler amount, with two sizes of nanoparticles, namely 12 nm (silica) and ~100 nm (Cu-MBGN), along with inert glass microfillers.

The trimodal filler mixture was apparently more successful than the bimodal approach. Cu-BG composites with silica (1-CuBG-Si, 5-CuBG-Si, and 10-CuBG-Si) kept high FS values after 28 days and were statistically similar to values after 1 day. The reinforcing effect of Cu-MBGN particles could be ascribed to a combination of resin interlocking in the porosities of Cu-MBGN and additional reinforcing effect of silica, which prevented composite deterioration in water after 28 days. On the other hand, a drop of FS in the inert control 15-Si could be related to silane water degradation, leading to hydrolytic degradation and resin plasticization [13].

Similar to the bimodal approach, in CuBG composites with silica, the gradual replacement of silica with Cu-MBGN also provided a higher volume and higher FM and MH, thus retaining the favorable stiffness and hardness. The results are in agreement with other studies [18,31,32].

All the tested materials achieved high DC values except for 10-CuBG-Si. The high surface area of mesoporous nanoparticles limits their addition to resin. A higher amount of resin is necessary to wet each particle, leading to a steep increase in the composite’s viscosity with higher Cu-MBGN inclusion. Uniform dispersion of nanoparticles becomes increasingly difficult with higher amount. If unsuccessful, particles group together due to weak van der Waals attractive forces [26,31]. Material 10-CuBG-Si had the highest volume filler loading in this study. Furthermore, the material had poor handling properties and was visually very dry. Significant packing was necessary to produce satisfactory specimens. The consequences of this disadvantage were not apparent in the tested properties, except for DC. The 35 wt % of resin was not sufficient to ensure adequate radical mobility and complete polymerization of 10-CuBG-Si. In contrast, the favorable viscosity of bimodal 10-CuBG probably contributed to the highest DC of all the tested materials. Unsilanized Cu-MBGN did not limit the mobility of the radicals, leading to a higher number of reacted C=C bonds [35].

A key to uniform dispersion of nanoparticles in general, and especially mesoporous particles, in resin composites is the mixing process. Numerous techniques have been developed to overcome this obstacle [26,31]. A high-temperature, high vacuum mixing process was proposed by Samuel et al. [18]. An unfilled resin matrix was heated to 80 °C to reduce its viscosity and achieve penetration of organic monomers into porosities at the surface of filler particles. Additionally, vacuum was used to release trapped air inclusions and facilitate the flow into porosities. In that study, mesoporous particles had relatively large porosities of 0.46 cm^3^/g with a 50% volume fraction [18]. Although the resin was preheated in this study, there is still room for improvement.

Trimodal filler mixture, in particular materials with 1 and 5% of Cu-MBGN, demonstrated high polymerization, strength, modulus, and hardness.

## 5. Conclusions

The bimodal combination of Cu-MBGN and microfillers elevated the stiffness and hardness of the resin composites while decreasing strength. However, trimodal CuBG composites with silica and microfillers demonstrated durable strength after 28 days and increased elastic modulus and hardness, with very high polymerization efficiency. Due to these characteristics, the trimodal filler mixture appears to be a promising path for future investigations of antibacterial properties and ion release. Within the limitations of this study, we expect that the incorporation of Cu-MBGN as an addition to inert silica and glass microfillers in dental resin composite could result in an effective and resistant remineralizing restorative material.

## Figures and Tables

**Table 1:** Characteristics of fillers used in the present study. Data provided by the manufacturers.

Name	Type	Manufacturer/ Product	Composition (wt %)	Size	Silanization
Cu-MBGN	Experimental/ Bioactive	Laboratory made	SiO_2_ 84.8% CaO 9.4% CuO 5.8% *	~100 nm	No
45S5 bioactive glass	Commercial/ Bioactive	Schott, Germany G018-144	SiO_2_ 45% Na_2_O 24.5% CaO 24.5% P_2_O_5_ 6%	4.0 µm	No
Ba glass	Commercial/ Inert	Schott, Germany GM27884	SiO_2_ 55.0% BaO 25.0% B_2_O_3_ 10.0% Al_2_O_3_ 10.0%	1.0 µm	Yes 3.2%
Silica	Commercial/ Inert	Evonik Degussa, Germany Aerosil DT	SiO_2_ > 99.8%	12 nm	Yes 4–6%

* composition determined by ICP-AES analysis, data from [24].

**Table 2:** Composition of experimental resin composites (all amounts in wt %).

Group	Material	Resin	Inert Microfillers	Silica Nanofillers	Cu-MBGN	45S5 BG
Bimodal approach (65% filler load)	10-CuBG	35%	55%	-	10%	-
	10-BG			-	-	10%
	10-Si			10%	-	-
Trimodal approach (70% filler load)	1-CuBG-Si	30%	55%	14%	1%	-
	5-CuBG-Si			10%	5%	-
	10-CuBG-Si			5%	10%	-
	15-BG			-	-	15%
	15-Si			15%	-	-

## Data Availability

The datasets generated and analyzed in the present study are available from the corresponding author on reasonable request.
